# Detection of Mycoplasma Contamination of Cell Culture by A Loop-Mediated Isothermal Amplification Method

**DOI:** 10.22074/cellj.2019.5624

**Published:** 2018-11-18

**Authors:** Zohre Soheily, Mohammad Soleimani, Majidzadeh- Ardebili Keivan

**Affiliations:** 1 Department of Microbiology, Qom Branch, Islamic Azad University, Qom, Iran; 2 Department of Microbiology, Faculty of Medicine, AJA University of Medical Sciences, Tehran, Iran; 3 Tasnim Biotechnology Research Center (TBRC), Faculty of Medicine, AJA University of Medical Sciences, Tehran, Iran; 4 Motamed Cancer Institute, ACECR, Tehran, Iran

**Keywords:** Cell Culture, Loop-Mediated Isothermal Amplification, Mycoplasma, Polymerase Chain Reaction

## Abstract

**Objective:**

Mycoplasmas are major contaminants of cell culture and affect *in vitro* biological and diagnostic tests. Mycoplasma detection is conducted using culture and molecular methods. These methods vary in terms of accuracy, reliably and sensitivity. Loop-mediated isothermal amplification (LAMP) is used to amplify target DNA in a highly specific and rapid manner. This study aimed to develop a LAMP method for rapid detection of Mycoplasma in culture samples.

**Materials and Methods:**

In this descriptive laboratory study, for LAMP detection of Mycoplasma contaminations in cell culture, we used primers specifically designed for targeting the 16S rRNA conserved gene of *Mycoplasma* spp. For a positive control structure, 16S rRNA amplified based on PCR, was cloned in a plasmid vector and sequenced. The assay specificity was evaluated using Mycoplasma genomic DNA and a panel containing genomes of gram-positive and gram-negative organisms.

**Results:**

In this study, the method developed for detection of Mycoplasma contamination of cell cultures was a rapid, sensitive and cost-effective LAMP approach. The results demonstrated that this method benefits from high specificity (100%) for amplification of Mycoplasma strains and high speed (multiplication within 60 minutes), while it does not require expensive laboratory equipment compared to those needed for polymerase chain reaction (PCR)-based detection.

**Conclusion:**

Our study is the first report about application of LAMP assay based on 16S rRNA gene for detection of Mycoplasma strains; this technique could be considered a useful tool for rapid detection of contamination of cell culture.

## Introduction

Over the last decades, cell culture has been frequently used as a main research tool in medical and biological experiments (1). Cell culture contaminants can be categorized into chemical and biological contaminants. Impurities in media, and sera, endotoxins, and detergents are major chemical contaminants, and bacteria, molds, yeasts, viruses, mycoplasma, and cross contamination of other cell lines, are regarded as biological contaminants (2). Mycoplasma contamination is a serious concern that exists when using cell culture (3, 4). The primary starting material, glassware or apparatus, culture reagents (mainly fetal bovine serum), laboratory staff and cross- contamination of infected cultures are examples of Mycoplasma contamination sources (5, 6). The prevalence of Mycoplasma contamination of cell cultures has been estimated to range from 5 to 35% (7, 8) while prevalence of cell culture infections with two or more Mycoplasma species are between 7 and 60% (1). Mycoplasma infection affects different aspects of the infected cell culture, resulting in obtaining spurious experimental data (5, 9). Mycoplasmas are the smallest free-living microorganisms which are characterized by their round or filamentous shape, absence of a rigid cell wall and a DNA genome in the Mb range (4, 7, 10). Most of Mycoplasma species are not pathogenic (7); however, *Mycoplasma pneumoniae* is a human pathogen (11). The species that are frequently found in cell culture are *Acholeplasma laidlawii, Mycoplasma arginini, Mycoplasma fermentans, Mycoplasma hominis, Mycoplasma hyorhinis*, and *Mycoplasma orale* (12). 

Several methods have been developed for detection of *Mycoplasma* spp. (13) including microbiological cultivation, biochemical assays, ELISA, direct or indirect fluorescent staining, immunofluorescence and nucleic acid amplification techniques [direct or nested polymerase chain reaction (PCR)] (7, 9). Isolation on selective microbiological growth media has been regarded as the reference method as well the ‘gold standard’ assay, for a long period of time (14). Unfortunately, routine diagnosis procedures are usually time-consuming (i.e. several weeks are required to achieve results) and need high-level technical skills and expert personnel. Thus, fast and sensitive detection methods are needed to evaluate putative contaminated cell cultures. In this regard, newer test systems developed based on molecular biological methods, in particular PCR, which give results within 4.5-24 hours are commonly used by cell culture laboratories (15). However, complicated procedures and relatively costly machinery required for PCR and electrophoresis processes have restricted its use (16, 17). Therefore, development of simple, sensitive, specific, rapid and low- cost detection methods is of crucial importance (11).

Loop-mediated isothermal amplification (LAMP) assay is a novel gene amplification technique which uses 4-6 primers that recognize specific regions on the target DNA. The LAMP reaction is carried out under isothermal conditions (60-65°C), thereby obviating the need for a thermal cycler (18, 19). This method amplifies specific sequences of DNA in a shorter period of time compared to PCR with high specificity and efficiency but no need for a special reagent (17). Moreover, the LAMP reaction’s product can be detected in real time by turbidity monitoring, which the turbidity is correlated with the production of magnesium pyrophosphate (20), or optical monitoring of a fluorescent intercalating dye by naked eyes (21). Therefore, it can be used for a rapid detection of various infectious diseases (18, 22).

The aim of this study was to design and develop a reliable, rapid and specific LAMP assay based on the conserved section of 16S rRNA gene for detection of Mycoplasma contamination of cell cultures. To the best of our knowledge, this is the first report on application of this method for detection of *Mycoplasma* contamination in cell cultures based on 16S rRNA gene.

## Materials and Methods

### Primer design

Initially, 16S rRNA sequences of *Mycoplasma* spp. were retrieved from GenBank (http://www.ncbi.nlm. nih.gov/genbank/). These sequences were selected from 12 *Mycoplasma hominis*, 8 *Mycoplasma hyorhinis,* 3 *Mycoplasma salivarium,* 3 *Mycoplasma orale*, 3 *Acholeplasma laidlawii,* 1 *Mycoplasma arginine,* 5 *Mycoplasma fermentans*, and 5 *Spiroplasma*. The sequences were aligned using CLC Sequence Viewer 6.4 (CLC bio, Aarhus, Denmark). Then, a set of six Mycoplasma-specific LAMP primers containing outer primers (F3-Myco and B3-Myco primers), inner primers (FIP-Myco and BIP-Myco) and a loop primer (loop- Myco) were designed based on the consensus sequence of the target gene by an online software program (Primer Explorer V4) from Eiken chemical (http://primerexplorer. jp/e/). The theoretical specificity of the designed primers was checked by an in-silico analysis using BLAST and Primer-BLAST on NCBI Server (http://www.blast.ncbi. nlm.nih.gov/). The LAMP primers were synthesized commercially (Bioneer, Korea) (Table 1).

### Cell culture and bacteria and DNA extraction

To perform this descriptive laboratory study, ten cell cultures contaminated with mycoplasmas, some non- contaminated cell culture and a DNA reference standard Mycoplasma were obtained from the Academic Center for Education, Culture and Research of Tehran, Iran. The standard bacteria including *Shigella sonnei* ATCC 9290, enteropathogenic *Escherichia coli* (EPEC) ATCC 43887, *Klebsiella pneumoniae* ATCC 7881, *Bacillus subtilis* ATCC 6051, *Pseudomonas aeruginosa* ATCC 9027, *Staphylococcus aureus* ATCC 25923, *Enterococcus faecalis* ATCC 29212, and *Yersinia enterocolitica *ATCC 23715, were used as negative control in specificity testing. DNAof all cell cultures and standard bacterial species were extracted using the EZ-10 Spin Column Genomic DNA kit (Bio Basic Inc., Ontario, CA) according to the manufacturer’s instructions. DNA was quantified spectrophotometrically and then stored at -20°C till used as PCR template DNA and in LAMP assay and specificity tests.

### Polymerase chain reaction reactions

Amplification reaction was performed for DNA of contaminated cell cultures, non-contaminated cell cultures, *Mycoplasma* DNA reference and negative control strains in 25 µl volume containing 6 µl of purified DNA, 12.5 µl of 2X reaction mix, 0.5 µM of each F3Myco and B3-Myco outer primers (Table 1), 1 U of Taq DNA polymerase and 4.5 µl double distilled water. The PCR conditions were as follows: After an initial denaturing step at 94°C for 4 minutes, 35 cycles of the following steps were carried out: denaturation at 94°C for 45 seconds; annealing at 48.1°C for 45 seconds, and extension at 72°C for 45 seconds. Thermal cycling was carried out using an ABI 2720 thermocycler (Applied Biosystems, Warrington, UK). PCR products were separated on 2% agarose gels and compared against 100 bp DNA ladder (Fermentas, Lithuania) as a size marker, under UV gel documentation. 

**Table 1:** Primers for 16S rRNA gene of Mycoplasma spp. used in the loop-mediated isothermal amplification and polymerase chain reaction


Sequence (5′–3′)	Primer

*F3-Myco*	GCG ATG GCT AAC TAT GTC CC
*B3-Myco*	TCG CCT TTG GTG TTC TTC C
*FIP-Myco*	AGC CTA CGA ACG CTT TAC GCC CAG CCG TAA TAC ATA GG
*BIP-Myco*	AAC CCT GGC TCG CTT TGG ATA CGC ATT TCA CCG CTT CA
*LOOP-Myco*	CAA TAA TTC CGG ATA ACG CTT GC


### Cloning and preparation of standard plasmid 

After PCR amplification of the 16S rRNA gene of *Mycoplasma* using the outer primers, TA cloning of the product was performed. For this purpose, the PCR product was purified using the PCR Purification Kit (Bioneer, Korea). The purified 16S rRNA gene fragment with the length of 219 bp was ligated into pTZ57R/T vector by 1 U of T4 DNA ligase, according to instructions of InsTAcloneTM PCR Cloning Kit (Fermentas, Lithuania). Competent cells of E. coli Top10 F´ were transformed with the ligation reaction product. The transformed cells were incubated at 37°C for 24 hours on Luria-Bertani (Merck, Germany) medium containing 38.4 µg/ml IPTG (isopropylbeta- D-thiogalactopyranoside, Sigma, St. Louis, MO, USA), 40 µl/ml X-gal (5-bromo-4-chloro-3indolyl beta D-galactoside, Sigma, Germany), 50 µg/ ml nalidixic acid and 100 µg/ml ampicillin (Merck, Germany). Recombinant clones on the medium were identified by blue/white screening and some white colonies containing recombinant vector were chosen for extra evaluation. Then, plasmids of the selected clones were extracted by AccuPrep Plasmid Mini Extraction kit (Bioneer, Korea) and 16S rRNA gene containing recombinant plasmids, were confirmed by PCR with the outer primers and sequencing. The confirmed plasmid was named pTZ57R/T-16S rRNA and quantified using UV absorbance measurement at 260 and 280 nm and further used as positive control in the LAMP assay. 

### The loop-mediated isothermal amplification assay

The LAMP reaction was conducted for all *Mycoplasma* DNA extracted from cell culture samples in 25 µl reaction mixture containing 12.5 µl 2X reaction mix, 1.5 µl the primer mix (40 pmol of each inner primer and 5 pmol of each outer primer) (Table 1), 8 U of Bst DNA polymerase large fragment (New England Biolabs, Ipswich, MA, USA), 6 µl of template genomic DNA and 5 µl molecular grade water. The mixture was incubated at 63°C for 60 minutes in a Loopamp real- time turbidimeter (LA-320C, Teramecs, Japan), and turbidity of the reaction mix was determined at 650 nm every 6 seconds. Finally, the reaction was terminated by heating to 80°C for 5 minutes in order to denature the Bst DNA polymerase large fragment. The LAMP reactions were examined by Loopamp real-time turbidimeter, electrophoresis of products on 2% agarose gel and direct visual observation to judge turbidity. Cycle sequencing method using F3-Myco and B3Myco primers, was performed for final confirmation of the amplified products. The sequencing results were checked by BLAST (http://blast.ncbi.nlm.nih.gov/ Blast.cgi). To determine the optimum temperature that should be considered for LAMP reactions, the LAMP reactions were also performed at temperatures 60 and 65°C for 60 minutes. In order to assess the effect of the loop primer on amplification speed, 20 pmol of the loop primer (LOOP-Myco) was added to the reaction mixture and LAMP reaction was conducted after 30, 45 and 60 minutes. The negative control tubes (without template DNA) were included in each run. 

### Specificity and sensitivity of the loop-mediated isothermal amplification assay

For evaluation of the specificity of the test, the LAMP reactions were performed (based on the above-noted protocol) using genomic DNA of *Mycoplasma* spp. and also genomic DNA of non-Mycoplasma organisms (negative control bacteria). The reactions were assessed with naked eye inspection and the Loopamp real-time turbidimeter. In addition, electrophoresis on 2% agarose gel was carried out to confirm the results.

Moreover, 10-fold serial dilutions of pTZ57R/T-16S rRNA plasmid (135 ng to 0.135 fg equal 4×10^10^ to ~4 copies) were applied in LAMP experiment to examine the sensitivity of the assay. The results of amplified target sequence were analyzed by using Loopamp real-time turbidimeter, visual observation of turbidity by naked eye and electrophoresis on 2% agarose gel. Finally, sensitivity or detection limit (LOD) of the assay was determined.

## Results

### Analysis of the polymerase chain reaction products and cloning

**Fig.1:**
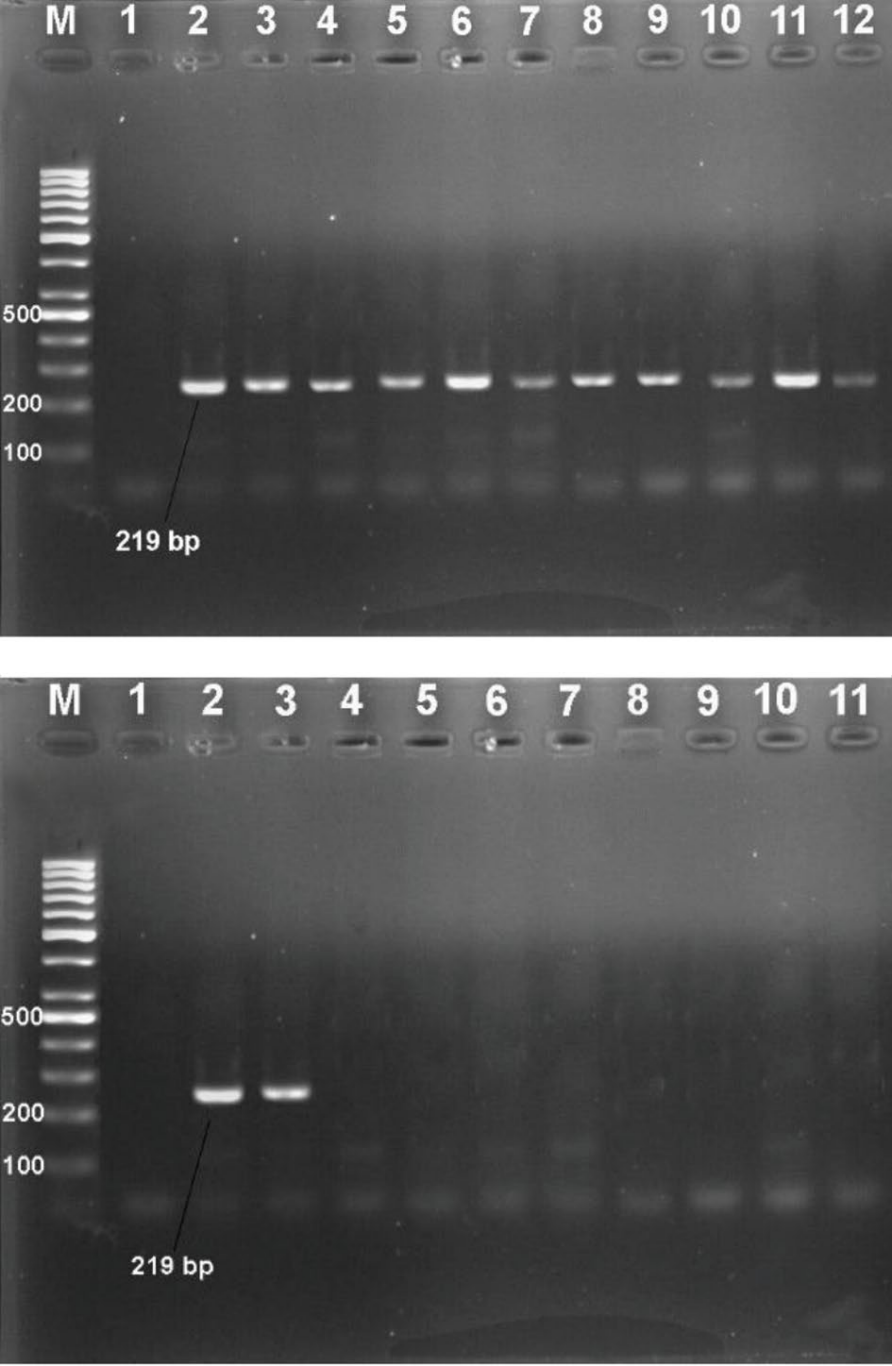
Polymerase chain reaction (PCR) experiments on contaminated cell cultures and negative control samples. A. Agarose gel electrophoresis of 16S rRNA PCR products (~ 219 bp) of Mycoplasma by using F3-Myco and B3-Myco primers. Lane M; 100 bp DNA ladder, Lane 1; Negative control, Lane 2; Standard Mycoplasma, Lanes 3-12; Positive amplification of contaminated cell cultures and B. Specificity of the 16S rRNA Mycoplasma PCR. Lane M; 100 bp DNA ladder, Lane 1; Negative control, Lane 2; Standard Mycoplasma, Lane 3; Contaminated cell culture, Lane 4; *Shigella sonnei* ATCC 9290, Lane 5; *Escherichia coli* ATCC 43887, Lane 6; *Klebsiella pneumoniae* ATCC 7881, Lane 7; *Bacillus subtilis* ATCC 6051, Lane 8; *Pseudomonas aeruginosa* ATCC 9027, Lane 9; *Staphylococcus aureus* ATCC 25923, Lane 10; *Enterococcus faecalis* ATCC 29212, and Lane 11; *Yersinia enterocolitica* ATCC 23715.

The PCR reaction was carried out using F3-Myco and B3-Myco primers on DNA of contaminated cell cultures, non-contaminated cell cultures, Mycoplasma DNA reference and negative control strains. PCR products of the tubes containing *Mycoplasma* DNA showed about 219 bp bands on 2% agarose gel. Based on comparison made against 100 bp DNA ladder, no significant difference found in banding pattern compared to the reference *Mycoplasma* strains (Fig .1A). The PCR reaction was specific as it showed exclusive amplification for *Mycoplasma* spp. while this result for 8 non-Mycoplasma bacteria species was negative (Fig .1B). Confirmatory test based on the cloning process was conducted by PCR assay with the outer primers (F3 and B3) on the extracted recombinant 16S rRNA-plasmids from white colonies and as expected, a clear and sharp 219 bp band on agarose gel was observed. In addition, the sequence of the amplified 16S rRNA gene was confirmed through direct sequencing, in which the obtained sequences perfectly matched the expected DNA sequences (data not shown). 

### Analysis of the loop-mediated isothermal amplification reaction

**Fig.2:**
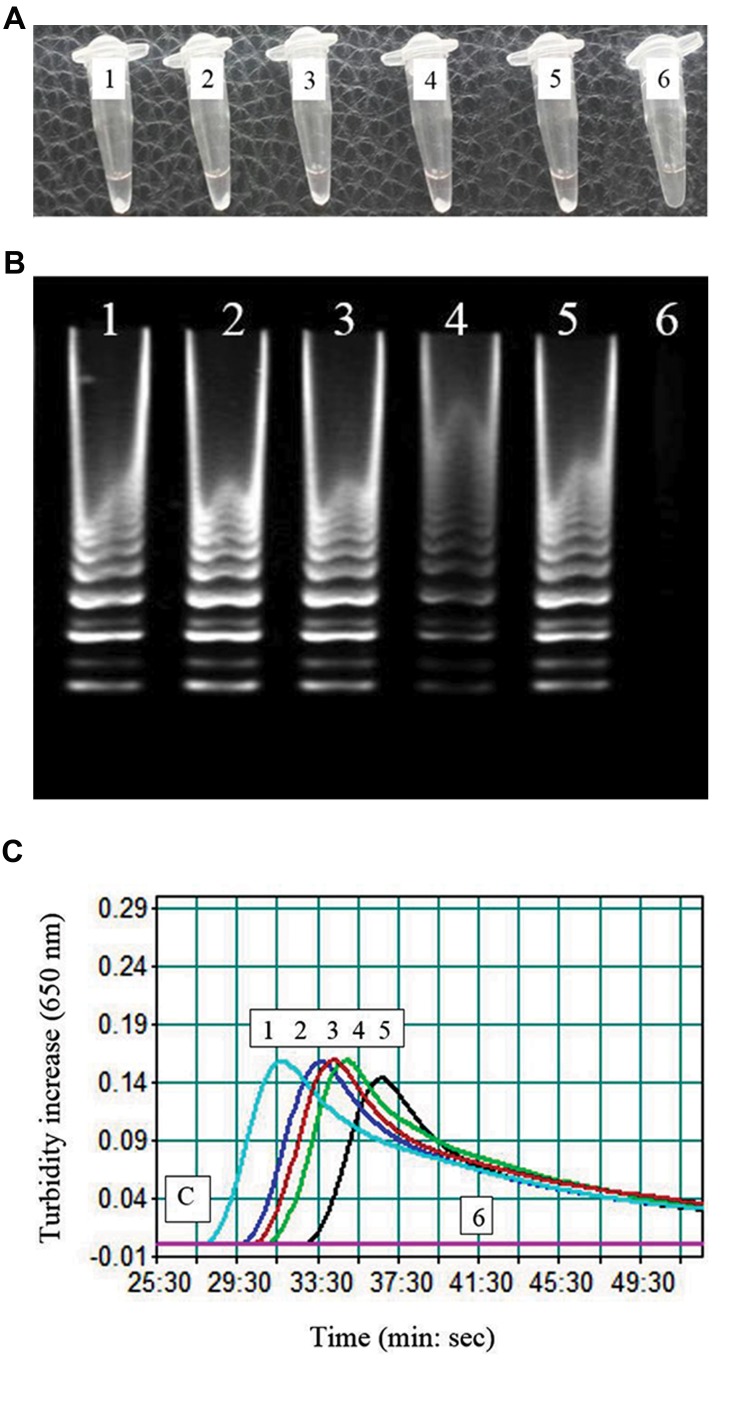
Loop-mediated isothermal amplification (LAMP) experiments on contaminated cell cultures. A. Visual appearance of the LAMP reactions. Showing white turbidity, the tubes 1-5 (contaminated cell culture samples) were positive, while the tube 6 was negative, B. Electrophoretic analysis of the LAMP products. In lanes 1-5, contaminated cell culture samples showed ladder-like pattern, lane 6 was negative control and had no ladder-like pattern, and C. A representative turbidity amplification graph of the LAMP reaction. Curves 1-5 represent contaminated cell cultures and curve 6 is for negative control.

In the tubes with positive reaction for isothermal amplification, the turbidity (caused by white magnesium pyrophosphate precipitation) was observed with naked eye (Fig .2A). Electrophoresis of the LAMP products on 2% agarose gel showed clear ladder-like DNA amplification (Fig .2B). Amplification graph of the real-time turbidimeter confirmed amplification of the 16s rRNA gene (Fig .2C). The specificity of the LAMP products was verified by cycle sequencing. Comparison of the sequencing results with the 16S rRNA sequences of *Mycoplasma spp*. in Gene bank database confirmed the validity of the products. Amplification was detected at 60, 63, and 65°C, and showed higher levels of amplified DNA at 60°C when compared to other temperatures. Also, effect of the loop primer (LOOP-Myco) on diminution of incubation time was explored. In the reactions without the loop primer, ideal time for isothermal amplification was 60 minutes while in the reaction containing the loop primer, it was 30 minutes. 

### Specificity and sensitivity of the loop-mediated isothermal amplification assay

The LAMPassay was specific because judgment graph of the real-time turbidimeter showed exclusive amplification for *Mycoplasma* spp. while 8 non-Mycoplasma bacteria species had negative results. Consistently, *in silico* analysis using BLAST, indicated that there were no false- positive nor false-negative amplification. In addition, gel agarose electrophoresis of the LAMP products showed the characteristic ladder-like multiple bands only in the tubes containing *Mycoplasma* spp. genome DNA. Inspecting the judgment plot, agarose gel electrophoresis and visual detection of turbidity, the LOD of the assay was found ~4000 copy per reaction tube.

## Discussion

Contamination of cell cultures by *Mycoplasma* spp. is a main problem in cell culture for which an accurate diagnostic method is highly required. There are several conventional and molecular diagnostic techniques available for detection of *Mycoplasma* spp. (9, 14). In the last decade, in Iran, several studies established PCR assays using 16S rRNA gene for detection of different species of mycoplasmas such as M. orale (23), *Mycoplasma* and *Ureaplasma* species (24), *Mycoplasma* spp. (25-27), in cell culture. The results of these papers demonstrated that 16S rRNA-based PCR could detect all common Mycoplasma that contaminate cell cultures. The findings of the current study are in agreement with those reported by Tang et al. (9) which confirmed that 16S rRNA is a suitable target for *Mycoplasma* detection using PCR; however, few cross-reactions were observed with closely related Gram-positive organisms. In addition, Molla Kazemiha et al. (28) showed that real-time PCR and PCR assays developed based on the public sequences in the 16S rRNA, are suitable methods with high sensitivity, specificity and accuracy for detection of mycoplasma contamination of cell cultures. However, these molecular methods are complex and time-consuming and they pose a risk of contamination with ethidium bromide that requires expensive apparatus and qualified laboratory technicians.

As LAMP method has several advantages benefits including no need for a special process, completion of the reaction in a single tube and approval by naked eye contrary to electrophoresis, it can be a suitable alternative for techniques used for *Mycoplasma* detection (29). Another advantage of the LAMP method is the high stability of Bst polymerase enzyme compared to a number of inhibitory factors such as EDTA, bile salts, and NaCl in the amplification reaction (30, 31). These characteristics show that utilization of the LAMP assay in laboratories with limited equipment and in large scale, is valuable. Yoshino et al. (32) showed the same sensitivity and specificity when comparing LAMP assay with a PCR assay, for rapid detection of *M. pneumoniae*. Also, a good global agreement between the LAMP assay and serological results for *M. pneumoniae* detection in pediatric patients, was revealed by Gotoh et al. (11). 

Our study defines a rapid, sensitive and cost-effective LAMP method which is comparable to other DNA amplification procedures that are extensively used for identification of various microorganisms in laboratory. Previously, various sequences in the genome of human pathogenic species of *Mycoplasma* were used in the LAMP assay for detection of different species belonging to this genus, such as *pdhD* gene of *M. genitalium* (21), *mhp165* gene of *M. hyopneumoniae* (17), uvrC gene of *Mycoplasma bovis* (33), p36 gene of Mycoplasma hyopneumoniae (34), the SDC1 sequence (M35024) of *Mycoplasma pneumoniae* (11, 32, 35), and P1 adhesin gene of *M. pneumoniae* (36). We used the 16S rRNA gene which is highly conserved in all *Mycoplasma* species and is the best target for genus-level detection of *Mycoplasma* spp. (37). Our finding demonstrated that ideal time and temperature for isothermal amplification was at 60°C in 60 minutes while Davudi-Asl optimized the LAMP test using the large Bst enzyme fragment at 66°C for 1 hour (36).

The primers designed in this study were theoretically completely specific for the 16S rRNA gene of *Mycoplasma*. Therefore, amplification was carried out only with DNA of *Mycoplasma*; also, neither false- positive and false-negative results in the LAMP assay nor any cross-reactivity with other species was observed. In addition, the sequencing results were in accordance with deposited sequence of 16S rRNA in NCBI. These results demonstrated that this technique has high specificity (100%) for the amplification of *Mycoplasma* strains and detected it with high efficiency. It seems that the extremely high specificity of the LAMP method is a result of using four primers that recognize distinct regions on the target sequences (38). Furthermore, using the designed loop primers in the mixture could increase rapidity and efficiency of amplification by attaching to the stem loops formed during reaction process (39). In our study, the LAMP assay was able to detect *Mycoplasma* DNA extracted from culture medium which approves this assay for detection of *Mycoplasma* strains in cell culture samples; with the help of such assays, *Mycoplasma* infection can be discovered at an early stage.

## Conclusion

Our study is the first report about designing and developing a LAMP assay based on 16S rRNA gene for determination of *Mycoplasma* strains contaminating cell culture. Based on our findings, LAMP assay developed based on 16S rRNA gene is highly suggested as a useful tool for rapid diagnosis of common *Mycoplasma* which contaminate cell culture. 
